# In vivo whole-cell recording with high success rate in anaesthetized and awake mammalian brains

**DOI:** 10.1186/s13041-016-0266-7

**Published:** 2016-09-29

**Authors:** Yao Wang, Yu-zhang Liu, Shi-yi Wang, Zhiru Wang

**Affiliations:** Institute and Key Laboratory of Brain Functional Genomics of Chinese Ministry of Education, Shanghai Key Laboratory of Brain Functional Genomics, School of Life Sciences, East China Normal University, Shanghai, 200062 China

**Keywords:** In vivo, Whole-cell recording, Amphotericin, Anaesthetized, Awake, Cortex, Hippocampus

## Abstract

As a critical technique for dissection of synaptic and cellular mechanisms, whole-cell patch-clamp recording has become feasible for in vivo preparations including both anaesthetized and awake mammalian brains. However, compared with in vitro whole-cell recording, in vivo whole-cell recording often suffers from low success rates and high access resistance, preventing its wide application in physiological analysis of neural circuits. Here, we describe experimental procedures for achieving in vivo amphotericin B-perforated whole-cell recording as well as conventional (breakthrough) whole-cell recording from rats and mice. The success rate of perforated whole-cell recordings was 70―80 % in the hippocampus and neocortex, and access resistance was 40―70 MΩ. The success rate of conventional whole-cell recordings was ~50 % in the hippocampus, with access resistance of 20―40 MΩ. Recordings were stable, and in awake, head-fixed animals, ~50 % whole-cell patched neurons could be held for > 1 hr. The conventional whole-cell recording also permitted infusion of pharmacological agents, such as intracellular blockers of Na^+^ channels and NMDA receptors. These findings open new possibilities for synaptic and cellular analysis in vivo.

**Electronic supplementary material:**

The online version of this article (doi:10.1186/s13041-016-0266-7) contains supplementary material, which is available to authorized users.

## Introduction

Whole-cell patch-clamp recording is an important neuroscience technique. It allows the measurement of both subthreshold electrical activity and suprathreshold firing of a cell. In addition, the membrane potential of the recorded cell can be altered by current injection for determining the existence and properties of specific ion channels or receptors, as well as for determining the effect of neuronal activity on neural circuit functions such as activity-induced synaptic plasticity. Moreover, whole-cell recording permits infusion of pharmacological agents in the cell to conduct intracellular pharmacological studies or label cell morphology. Due to the distinct advantages, the technique of whole-cell recording is very powerful for investigating the intrinsic and synaptic properties of a neuron. In addition to its early application for recordings from in vitro preparations [1–4], whole-cell recording has been more recently applied to in vivo preparations [5]. Many groups have reported their findings with the use of in vivo whole-cell recording, as performed in anaesthetized animals [6–9] as well as in awake, head-fixed [7, 10, 11] or freely moving animals [9, 12], but the low success rate to achieve recordings and the high access resistance have prevented wide application of this method. In this study, we have developed experimental procedures that could overcome these problems, making whole-cell recording much more useful for in vivo studies of synaptic and cellular mechanisms of brain functions.

## Results

### Craniotomy and dura dissection with or without removal of subdural meninges

Similar to previous reports [7, 9, 12], we performed a small craniotomy (2―3 mm for rats, 1―1.5 mm for mice) to reduce respiration- and heartbeat-induced pulsation of the cortex. In our experiments, this craniotomy resulted in no visible pulsation (under the dissection microscope at ⨯63 magnification) of the cortex, allowing stable whole-cell recordings.

We further found that the dissection of the dura mater or subdural meninges within the craniotomy is also a very critical procedure. For a high success rate of good whole-cell recordings (particularly the perforated whole-cell recording via the method described below), the dura mater should be dissected either without any mechanical damage to subdural meninges or with a complete removal of subdural meninges.

**Fig. 1:**
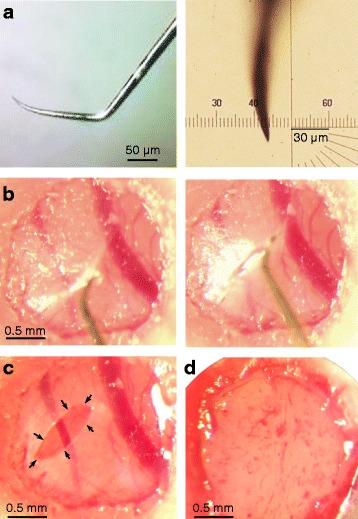
Dissection of the dura or subdural meninges. (**a**) Representative tip of the tungsten needle used for dissecting only the dura (the needle was bended as a hook), displayed with two different scales in left and right panels. (**b**) Dissecting the dura, during which the tungsten needle was used to hook up the dura and then remove it, and subdural meninges were not touched by the needle. (**c**) The cortical surface after dissecting the dura. (**d**) A representative cortical surface with the dura and subdural meninges completely dissected

To dissect the dura mater without any mechanical damage to subdural meninges, a tungsten needle with a sharp (<3 μm) and stiff tip (as illustrated in Fig. 1a) was used in our experiments. Such a needle was able to firmly hook up the dura, and then successfully penetrate and remove it without mechanically touching subdural meninges (as illustrated in Fig. 1b, c). In our observations, a mechanical touch by the dissection needle on subdural meninges (even without any visible damage under the dissection microscope at ⨯63 magnification) made the whole-cell recording (particularly perforated recording) very difficult to be achieved.

To dissect both the dura and subdural meninges, a tungsten or stainless steel needle with a relatively blunt tip (5―15 μm) and fine forceps were used after removing the dura for repeated removal of subdural meninges (see Fig. 1d for a representative cortical surface after dissecting completely subdural meninges). Because dissecting subdural meninges often caused bleeding, a complete dissection of these tissues usually took a long time (40―90 min). In addition, we found that any remaining subdural meninx above the cortical surface for pipette penetration made whole-cell recordings very difficult to be achieved. This is possibly because subdural meninges are soft, and when damaged, become sticky and should be completely removed to prevent contamination of the recording pipette as it penetrates the cortical surface.

Although both the dissection approaches (without touching subdural meninges and with complete removal of subdural meninges) allow for high success rates of good whole-cell recordings, we recommend the former approach, because it can be rapidly conducted and causes little bleeding and cell death in the cortex. However, some experiments (e.g., extracellular pharmacology by drug perfusion on the cortical surface) are likely to benefit from the latter approach.

### Requirement of a tight seal in the pipette pressure system

**Fig. 2:**
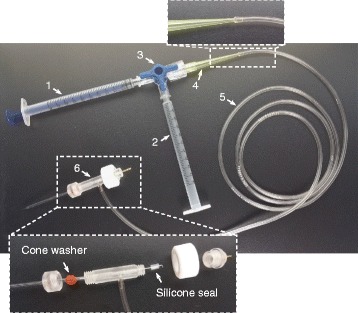
The pipette pressure system. (*1*) 1-ml syringe for applying positive pressure; (*2*) 1-ml syringe for applying negative pressure by the mouth (for seal formation, and in conventional whole-cell recording also for rupturing the cellular membrane); (*3*) three-way Stopcock; (*4*) 1-200 μl pipette tip; (*5*) suction tube (length, 110 cm); (*6*) pipette holder

We have also measured systematically the seal condition of the pipette pressure system that is required for high success rates of good recordings (the pressure system and its elements used in our experiments are shown in Fig. 2). We found that the pipette pressure system should be very tightly sealed, and for this purpose, some elements (particularly the 1-ml syringe for applying positive pressure, the three-way Stopcock, and the cone washer in the pipette holder) should be carefully selected and frequently replaced.

Our requirement for the seal condition of the pressure system is that when 950-mbar positive pressure is applied to the pipette back (in our experiments, 1-ml air was applied by the 1-ml syringe used for positive pressure), > 850-mbar positive pressure can be kept in the pressure system (with the three-way Stopcock closed) for at least 10 min (in our experiments, the sealed air was able to push the plunger of the 1-ml syringe for applying positive pressure back to the position at 0.8―0.85 ml when the three-way Stopcock was opened 10 min later). We found that only with such seal conditions, recordings with both a high success rate and good quality could be achieved.

### Use of glass beads in amphotericin internal solution for perforated whole-cell recording

Perforated whole-cell recording by including amphotericin B in internal solution is often used in in vitro studies, due to its advantages of stable access resistance and permeability only to monovalent ions but not to multivalent ions [13]. However, amphotericin solution contains insoluble precipitate, which may block the tip of recording pipettes. For in vitro recordings, a “tip filling” method has been reported to solve this problem [13]. In this method, amphotericin-free solution was backfilled in the pipette tip (with a distance of several hundred micrometers from the tip opening) before mounting the pipette in its holder for recordings. However, for in vivo recordings, because positive pressure is required to be applied to the pipette interior (high pressure is usually applied continuously during searching for cells), which can rapidly puff the precipitate in amphotericin internal solution to tip openings, the “tip filling” method does not appear to be suitable for avoiding the contamination of recording pipettes.

**Fig. 3:**
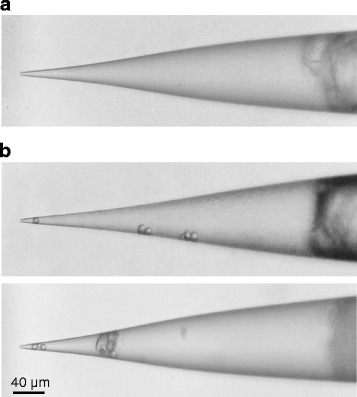
Recording pipettes containing glass beads in tips for perforated whole-cell recordings. (**a**) Before the application of air puff, a representative pipette showing the existence of precipitate in amphotericin internal solution. (**b**) Two representative pipettes showing the glass beads in their tips after the application of air puff

In a previously published study [14], we have shown our findings obtained by in vivo perforated whole-cell recording (in the rat visual cortex). Here, we describe in detail our method to achieve such recordings. In this method, a small amount of glass beads (5―15 μm in diameter) were added in amphotericin-containing internal solution and then evenly mixed with the solution. After filling the internal solution into the recording pipette, an air puff was applied to push one or several glass beads to the pipette tip (Fig. 3; also see Procedure), which was able to prevent the contamination of the pipette tip by precipitate in amphotericin solution. We further found that the best position of glass beads was 15―30 μm from the tip opening, and in our experiments the pipette resistance was increased from 1.6―2.0 MΩ to 3.5―4.0 MΩ.

### Achieving perforated whole-cell recording

**Fig. 4:**
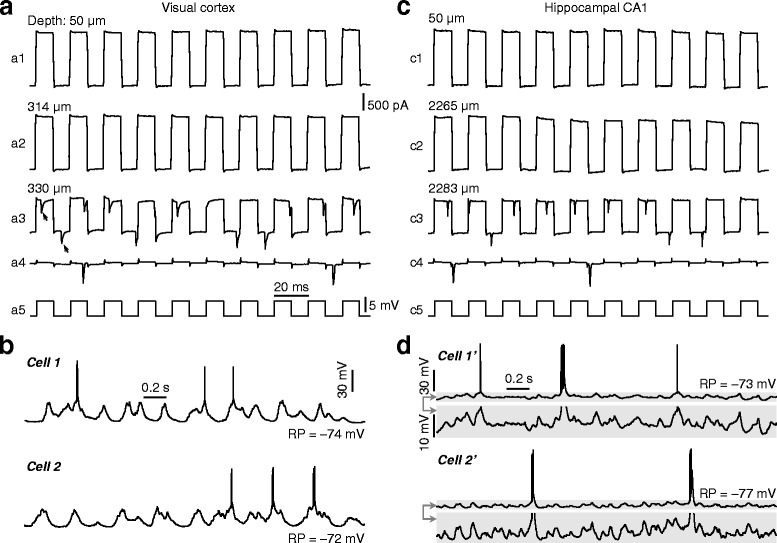
Achieving perforated whole-cell recordings from anaesthetized rats. (**a**) Perforated whole-cell recordings obtained in layer II/III of the visual cortex. Pipette resistances (monitored in voltage-clamp mode with the application of positive current pulses, as indicated in a5) before (*a1*, *a2*) and after (*a3*) the pipette had made contact with the neuronal membrane during searching for neurons, and pipette resistance after making seals (*a4*; the pipette potential was hyperpolarized to –70 mV for further seal formation). In (*a3*), note the AP-like inward currents (*as arrowed*) before making seals. The depths of the pipette tip beneath the cortical surface are indicated. (**b**) Spontaneous membrane-potential changes and spike activity of two example visual cortical neurons (in layer II/III) recorded in current-clamp mode (without current injection). The resting potential (RP; with a –13 mV liquid junction potential corrected) is indicated for each cell. (**c**, **d**) As in (**a**, **b**), for recordings in hippocampal CA1 pyramidal layers. Shadowed areas in (**d**) are displayed with two different scales

Then, “blind” perforated whole-cell recordings were made with the recording pipette that contained amphotericin solution as well as glass beads in its tip. Before the recording pipette was lowered down to the cortical surface, a high positive pressure of 250―350 mbar was applied to the pipette interior, and this pressure was kept in the pipette until it made contact with the neuron of interest. During searching for neurons in the brain, pipette resistance was monitored in voltage-clamp mode (at 0 mV, with the application of positive current pulses of 5 mV for 10 ms at 50 Hz). Pipettes were advanced in the brain at a speed of 15―30 μm/s, and the speed was reduced to 10―15 μm/s when pipettes arrived at the depth of interest. We found notably that immediately after the pipette had made contact with a neuronal membrane, both reproducible increase of pipette resistance (30―50 %) and action potential (AP)-like inward currents emerged (see Fig. 4a, c for two example recordings). Then, a gentle negative pressure was applied (by the mouth) to make seals to the cellular membrane, and the pipette potential was hyperpolarized to –70 mV for 1―2 min to facilitate seal formation (Fig. 4a, c).

**Fig. 5:**
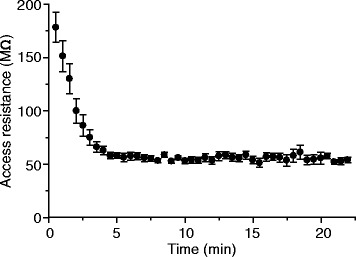
Time course of access resistance for perforated recordings. Recordings were made on neurons (*n* = 14) in the CA1 pyramidal layer of anaesthetized rats. Time 0, the time of initial seal formation. Results are plotted as mean ± SEM. The access resistance became stable ~5 min after seal formation

**Fig. 6:**
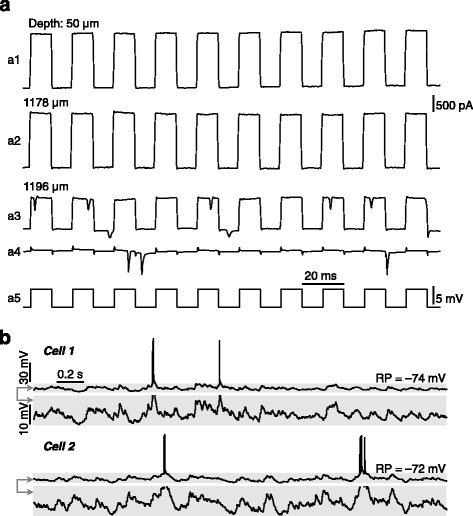
Achieving awake perforated whole-cell recordings. (**a**, **b**) In awake, head-fixed mice, achieving perforated whole-cell recordings (**a**) and spontaneous activity of two example awake recordings (**b**) from hippocampal CA1 pyramidal layers. Data are presented as in Fig. 4c, d

In our experiments, stable amphotericin perforation could be achieved ~5 min after initial seal formation (see Fig. 5 for the time course of access resistance of recordings made in the CA1 pyramidal layer from anaesthetized rats). In Fig. 4b, d, we show the spontaneous activities (when access resistance became stable) of representative perforated whole-cell recordings made in layer II/III of the visual cortex and in hippocampal CA1 pyramidal layers from anaesthetized rats. In addition, stable perforated whole-cell recordings could be achieved in awake, head-fixed mice via this method, and in Fig. 6, we show two representative awake recordings from CA1 pyramidal layers.

For the AP-like inward current that emerged when pipettes made contact with neuronal membranes (which was not seen in our conventional whole-cell recordings), our explanation is that amphotericin perforation took place immediately after a contact of the pipette had been made with the neuronal membrane (note the small, ~20 μm, distance between the depths at which AP-like currents emerged and pipette resistance had not been increased; as shown in Figs. 4a, c and 6a), making it possible to record neuronal firing (generated by the holding voltage and occurring spontaneously) before making seals.

On the other hand, when the pipette made contact with obstacles (e.g., blood vessels or glial cells) but not with neurons, there was only an increase in pipette resistance but not AP-like inward currents. Also, if the pipette pressure system was not sealed tightly enough (see the criteria described above), a contact with neuronal membranes resulted in only an increase of pipette resistance but no AP-like currents, and in such situations perforated whole-cell recordings could sometimes be successfully obtained. Nonetheless, we highly recommend using a tightly sealed pipette pressure system, because experimenters can easily determine whether the pipette has made contact with neurons or obstacles, and even when the contact is with neurons, the success rate of high-quality recordings can be much higher.

### Achieving conventional whole-cell recording

For “blind” conventional (breakthrough) whole-cell recordings, the dura or subdural meninges were dissected in the same way as for perforated recordings. There were three major differences between the conventional and perforated whole-cell recording methods. First, glass beads were not added in internal solution for conventional whole-cell recording. Second, a lower (60―80 mbar) positive pressure was applied to the back of recoding pipettes during searching for neurons in the brain (this pressure was kept in the pipette until it made contact with the neuron of interest). Third, when the pipette made contact with a neuronal membrane, there was only an increase in pipette resistance but not AP-like inward currents, which is similar to the conventional whole-cell recording reported by other in vivo studies [7, 9, 12].

**Fig. 7:**
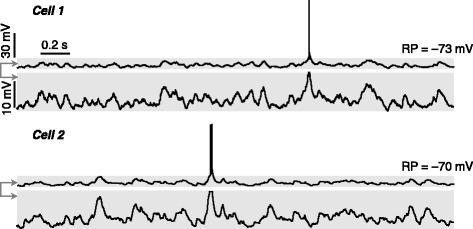
Representative conventional whole-cell recordings. Spontaneous activity of two example cells recorded in current-clamp mode (no current injection) from hippocampal CA1 pyramidal layers of anaesthetized rats. Shadowed areas are displayed with two different scales. For the resting potential (RP) of conventional whole-cell recordings, a –15 mV liquid junction potential was corrected

To search for neurons in the brain, recording pipettes were advanced at an initial speed of 15―30 μm/s, and then at a speed of 10―15 μm/s when reaching at the depth of interest. Once the pipette had made contact with neurons, 20―30 % increase of pipette resistance could be detected, and then a gentle negative pressure was applied (by the mouth) to achieve GΩ seals. The pipette potential was hyperpolarized to –70 mV for 0.5―1 min for further seal formation, after which a brief strong suction was applied (by the mouth) to achieve the whole-cell configuration. In Fig. 7, we show two representative conventional whole-cell recordings obtained from hippocampal CA1 pyramidal layers of anaesthetized rats.

### Intracellular loading of chemicals via conventional whole-cell recording

**Fig. 8:**
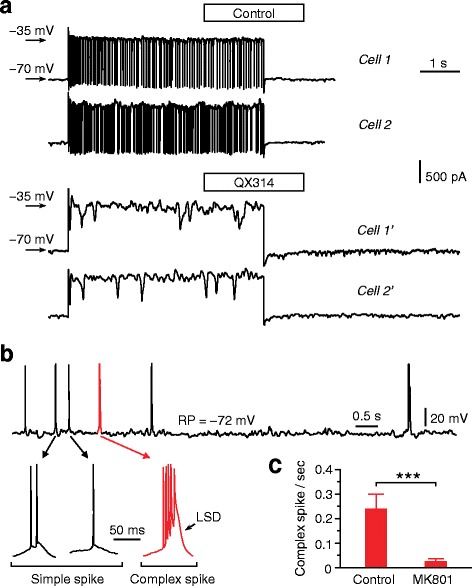
Intracellular pharmacology by conventional whole-cell recording. (**a**) Example cells showing that including QX314 in internal solution was able to block neuronal firing. Cells were recorded in voltage-clamp mode from CA1 pyramidal layers of anaesthetized rats. Without drug treatment (*two cells shown in top panel*), neuronal firing was elicited by depolarizing the cell to –35 mV (from –70 mV); in the presence of QX314 (*two cells shown in bottom panel*), neuronal firing could not be elicited by the same depolarization. (**b**, **c**) Blocking effect of intracellular application of MK801 on complex spikes that occurred spontaneously in neurons of CA1 pyramidal layers (data obtained from anaesthetized rats). Example traces (**b**; with low and high temporal resolutions shown in top and bottom panels, respectively) for simple (standard) spikes and complex spikes, which were recorded in the absence of MK801 (in current-clamp mode at resting potentials). Compared with simple spikes, complex spikes consisted of more (4―6) APs and a remarkably larger and slower depolarization (LSD). Summary of the frequencies of complex spikes recorded in the absence and presence of intracellular application of MK801 is shown in (**c**) (*n* = 18 each). ^***^ *P* < 0.001

The conventional whole-cell recording conducted in our experiments also permitted intracellular pharmacological studies. As observed in neurons of the CA1 pyramidal layer, including 2 mM QX314 (a blocker of Na^+^ channels) in the internal solution for conventional whole-cell recordings totally blocked the neuronal firing elicited by depolarizing the cell (Fig. 8a), and including 1 mM MK801 (a blocker of NMDA receptors) in internal solution greatly diminished complex spike bursts of CA1 neurons (Fig. 8b), which consisted of ≥ 4 (up to 6 in our recordings) APs as well as a remarkably larger and slower depolarization (compared with the depolarization with simple, standard spikes) and has been recently reported to depend on NMDA receptor-mediated activity [15].

### Access resistance, success rate, and recording stability

For perforated whole-cell recordings from anaesthetized rats, we used a quality criterion of resting potentials < –68 mV and spike amplitudes > 60 mV (up to 70 mV in our recordings). Access resistance of 40―70 MΩ (mean ± SD: 53 ± 10 MΩ) was found in patching neurons in the cortex and CA1 pyramidal layers. The success rate was ~80 % in patching cortical neurons and ~70 % in patching CA1 neurons. Once whole-cell patched, 70 % neurons could be held for > 1 hr.

In awake, head-fixed mice, perforated whole-cell recordings have been made on neurons in CA1 pyramidal layers, for which a quality criterion of resting potentials < –65 mV and spike amplitudes > 60 mV was used. Access resistance was 40―65 MΩ (mean ± SD: 51 ± 12 MΩ), and the success rate was ~70 % (both were similar to the recordings in anaesthetized rats). About 50 % awake perforated recordings could last for > 1 hr.

Conventional whole-cell recordings have been made on neurons in CA1 pyramidal layers from anaesthetized rats, for which we used a quality criterion of resting potentials < –70 mV and spike amplitudes > 70 mV (up to 90 mV in our recordings). Access resistance was 20―40 MΩ (mean ± SD: 30 ± 9 MΩ), and the success rate was ~50 %. About 60 % neurons could be held for > 1 hr.

## Discussion

In vivo whole-cell recording often suffers from the problems of low success rates and high access resistance. We found that for in vivo whole-cell recording including amphotericin B-perforated recording, several experimental procedures are of utmost importance: (1) The dura mater should be dissected either without any damage to subdural meninges (for this purpose, we suggest the use of a specific tungsten needle that has a sharp and stiff tip for dissection) or with complete removal of subdural meninges. (2) For perforated whole-cell recording, adding a small amount of glass beads in amphotericin internal solution can effectively avoid the contamination of the pipette tip by precipitate in amphotericin solution. (3) A very tight seal is required in the pipette pressure system, particularly for the perforated whole-cell recording (due to the existence of glass beads in pipette tips).

For an experienced experimenter, the success rate to obtain perforated whole-cell recordings and conventional whole-cell recordings in vivo can be expected to be 70―80 % and ~50 %, respectively (as performed in our experiments). In addition, the in vivo recording can be achieved with a satisfactory access resistance (40―70 MΩ and 20―40 MΩ in our perforated and conventional whole-cell recordings, respectively).

The conventional whole-cell recording can also permit infusion of pharmacological agents to block intracellularly ion channels and receptors (as shown in Fig. 8) as well as to label cell morphology (data shown in our unpublished study: Y. Wang and colleagues, manuscript submitted). In addition, recordings can be remarkably stable: in our experiments from anesthetized adult rats, ~70 % perforated whole-cell recordings and ~60 % conventional whole-cell recordings could last for > 1 hr; in our experiments from awake, head-fixed mice (adult), ~50 % perforated whole-cell recordings could last for > 1 hr. Besides our experiments that have been conducted in the neocortex and hippocampus, this recording technique may also be applied to some other brain regions.

However, there are still potential limitations in our technique. The major ones include: (1) the access resistance of conventional whole-cell recordings is not low enough to allow good voltage clamping, and (2) awake recordings cannot be achieved from freely moving animals. Thus, this technique remains to be further improved.

In summary, we have developed experimental procedures to overcome the problem of low success rates for achieving good whole-cell recordings from both anaesthetized and awake, head-fixed animals, which could make the whole-cell recording technique much more useful for synaptic and cellular analysis in in vivo mammalian brains.

## Methods

### Animals

All animal procedures were performed in accordance with the Animal Care and Use Committee of East China Normal University. Adult male rats (Sprague-Dawley, aged 10―14 weeks) and male mice (C57BL/6, aged 8―12 weeks) were used in this study.

### Animal preparation for recording under anaesthesia

Anaesthetized recordings were obtained from rats. Animals were anaesthetized with pentobarbital (80 mg/kg; i.p.). After tracheotomy, the head was restrained in a stereotaxic apparatus (David Kopf Instr.). The body temperature was maintained at 37.3―37.8 °C with a customized heating blanket, and 2―4 hr after initial anaesthesia, the anaesthesia level was constantly maintained with supplementary injections of pentobarbital (16―20 mg/kg/hr).

A small (2―3 mm diameter) craniotomy was made above the right cortex (from Bregma: 3.5―5.5 mm posterior and 2.5―3.5 mm lateral for recordings from the CA1 hippocampus; 6―8 mm posterior and 3―4.5 mm lateral for recording from the visual cortex). The dura mater or subdural meninges were then gently dissected, and the exposed brain surface was maintained moist with saline. For recording, the surface of the brain was cleaned with saline (using a small piece of cotton) before advancing the recording pipette into the brain. If the dura was dissected without removing subdural meninges (as shown in Fig. 1c) and the brain surface had not been cleaned for a long time period (>1―2 hr), an edge of the remaining dura (randomly selected) was hooked up by the tungsten needle (used for dissecting the dura) before cleaning the brain surface.

### Animal preparation for awake recording

Mice were used for awake recording. One week prior to recording, animals were anaesthetized with pentobarbital (85 mg/kg; i.p.). After cutting the scalp and cleaning completely the soft tissue above the skull, a customized metal plate allowing the exposure of Bregma and craniotomy was mounted on the skull with Vetbond tissue adhesive and dental cement. Animals were then returned to home cages. In the following days, animals were trained to get accustomed to head fixation, during which the metal plate mounted on the skull was screwed to a mental bar installed laterally on one side of the head, and animals were placed on a flat plate that could rotate smoothly around the center, similar to a recent study in the sensory cortex [11]. On the recording day, mice were head-fixed via the same procedure and anaesthetized with isoflurane (1.5 %). Craniotomy (1―1.5 mm diameter for mice) and dissection of dura or subdural meninges were performed as for recording under anaesthesia; animals were allowed to recover from isoflurane for > 1 hr (when normal behavioral activity occurred), during which the head was released. Recordings were conducted when the head was re-fixed, a session that lasted for 4―5 hours and consisted of one to three times of water application.

### Electrophysiology

Patch pipettes with a tip opening of 2.5―3.0 μm were pulled from borosilicate glass tubing (Kimble Glass Inc.), which had a resistance of 1.6―2.0 MΩ. For perforated whole-cell recordings, internal solution contained (in mM) 136.5 K-Gluconate, 17.5 KCl, 9.0 NaCl, 1.0 MgCl_2_, 10.0 HEPES, 0.2 EGTA, and amphotericin B (375 μg/ml); small amounts (0.5―0.8 mg/ml) of glass beads (5―15 μm in diameter) (Polysciences, Inc.) were included for the availability of the precipitate-free solution in pipette tips. For conventional whole-cell recording, internal solution contained (in mM) 125.0 K-Gluconate, 2.0 KCl, 10.0 Na_2_-phosphocreatine, 10.0 HEPES, 0.5 EGTA, 4.0 Mg-ATP, 0.3 Na_3_GTP.

Motor-driven manipulators (Siskiyou MMX7630, Siskiyou Corp.) were used for advancing recording pipettes. Signals were acquired with a patch-clamp amplifier (Axopatch 200B, Axon Instr.) and sampled at 5 kHz by a data acquisition card (Digidata 1440, Axon Instr.), with 1, 2, or 5 kHz low-pass filtering. Liquid junction potentials were corrected in this study: –13 mV for perforated and –15 mV for conventional whole-cell recordings.

After recording, stereotaxic coordinates of the cell were measured to determine the recording site. That the recording was obtained from hippocampal CA1 pyramidal layers was also indicated by the depth (rats, 120―220 μm; mice, 70―150 μm) of the recording site beneath the boundary between the cortex and the hippocampus, where a sudden jump of electrical signals could be seen by monitoring pipette resistance. In our unpublished study (Y. Wang and colleagues; manuscript submitted), we performed histological staining of neuronal morphology for recordings from CA1 pyramidal layers, and all neurons stained (*n* = 5 rats; *n* = 5 mice) were found to be pyramidal cells.

### Data analysis and statistics

Unless otherwise specified, statistical significance was determined using student’s *t*-test, and average values are presented as mean ± s.e.m.

## Materials

### Reagents

- Experimental animals: Adult Sprague-Dawley rats (male, aged 10―14 weeks) and C57BL/6 mice (male, aged 8―12 weeks). **!CAUTION** All animal studies must be approved by the Animal Care and Use Committee.
- Anesthetics: sodium pentobarbital (Merck) and isoflurane (Abott)
- Chemical components for internal solution (all from Sigma-Aldrich): K-gluconate (cat. no. G4500), KCl (cat. no. P5405), NaCl (cat. no. S5886), MgCl_2_ (cat. no. M0250), HEPES (cat. no. H3375), EGTA (cat. no. E4378), Amphotericin B (cat. no. A4888), DMSO (cat. no. D8418), Na_2_-phosphocreatine (cat. no. P7936), Mg-ATP (cat. no. A9187), Na_3_-GTP (cat. no. G8877), MK-801 (cat. no. M107), QX-314 (cat. no. L5783), KOH (cat. no. P5958) and HCl (cat. no. 304174)
- Glass beads (Polysciences, cat. no. 07666)
- Vetbond tissue adhesive (3 M Vetbond, cat. no. 1469SB)
- Dental acrylic (Pigeon Dental)

### Equipment

- Vortex mixer (Scientific Industries)
- Vibration isolation table (Technical Manufacturing Corporation)
- Customized faraday cage
- Animal body temperature control system (BASi, FHC-40908)
- Single Syringe Pump (New Era Pump Systems, NE-1000)
- Isoflurane inhalation system (Midmark, Matrx VMR)
- Dissection microscope (Olympus, SZX10)
- Inverted microscope (Nikon, TS100)
- Cold light source (World Precision Instruments, Z-LITE-Z)
- Stereotaxic apparatus (Stoelting, cat. no. 51600)
- Surgical tools (World Precision Instruments) **!CAUTION** All surgical tools should be cleaned and sterilized before surgery
- Customized metal plate and plate holder for awake, head-fixed recording (Fig. xx)
- Micro-drill (Fine Science Tools, cat. no. 19007-05) and micro-drill bur (tip diameter: 0.5―0.7 mm for mice, 0.6―0.8 mm for rats)
- Motorized micromanipulator (Siskiyou, MX7630) and axis digital readout (Siskiyou, DR1000)
- Tungsten needle (Fine Science Tools, cat. no. 10130-10 and 10130-05) and stainless steel needle (Fine Science Tools, cat. no. 26002-10 and 26002-20 ) for dissecting the dura and subdural meninges (the tungsten needle can also be customized)
- Three-way Stopcock for the pipette pressure system (Smiths Medical or Shanghai Shangyi Kangge Medical Equipment)
- Suction tube for the pipette pressure system (Tygon, R-3603)
- 1-200 μl pipette tip for the pipette pressure system (Quality Scientific Plastics, cat. no. T090-Q)
- Pipette holder for the pipette pressure system (Axon Instruments, cat. no. 1-HL-U)
- Borosilicate glass tubing (1.7―1.8 mm outer diameter, 0.2 mm wall thickness; Kimble Chase, cat. no. 34500-99) and low–resistance pipettes (1.6―2.0 MΩ ; tip size, 2.5―3.0 μm) pulled from the glass tubing for both perforated and conventional whole-cell recordings
- Pipette puller (Narishige, PC-10)
- Micro-forge (Narishige, MF-900)
- Patch-clamp amplifier (Axon Instruments, Axopatch 200B)
- Data acquisition system (Axon Instruments, Digidata 1440)

### Reagent setup

Internal solution for perforated whole-cell recordings (in mM): 136.5 K-Gluconate, 17.5 KCl, 9.0 NaCl, 1.0 MgCl_2_, 10.0 HEPES, and 0.2 EGTA. Internal solution for conventional whole-cell recordings (in mM): 125.0 K-Gluconate, 2.0 KCl, 10.0 Na_2_-phosphocreatine, 10.0 HEPES, 0.5 EGTA, 4.0 Mg-ATP, and 0.3 Na_3_GTP. The pH of internal solution was adjusted to 7.3.

To prepare the stock solution of amphotericin B, 3 mg amphotericin powder and 60 μl DMSO were added in a small centrifuge tube. This centrifuge tube was then placed on a vortex mixer for 3―5 min to dissolve amphotericin. The stock solution was separately stored in other centrifuge tubes (1.5 μl for each tube) at –20 °C (in dark condition) and used for recordings for no more than 1 week. Within 0.5―1 hr before achieving recordings, 0.2 ml internal solution was added in one of the centrifuge tubes that contained the stock solution (a final concentration of amphotericin was 375 μg/ml), and the centrifuge tube was placed on the vortex mixer for ~1 min to dissolve amphotericin in internal solution.

### Procedure

Check the seal condition of the pipette pressure system ● TIMING variable

1. Mount a recording pipette (no need to fill solution) in its holder of the pipette pressure system.
2. Apply 950-mbar positive pressure to the pipette back (in our pipette pressure system, as displayed in Fig. 2, 1-ml air was applied by the 1-ml syringe that was used for positive pressure), and then close the three-way Stopcock.
3. Ten minutes later, open the three-way Stopcock (with direction to the interior of the 1-ml syringe for positive pressure) to measure the remaining positive pressure. No lower than 850-mbar pressure should be kept in the system (in our pipette pressure system, the sealed air could push the syringe plunger back to the position at 0.8―0.85 mm); otherwise, replace some elements and reconnect the pressure system to test the seal condition.

? TROUBLESHOOTING (1)

▲CRITICAL This is one of the most critical steps for a high success rate of good patch recordings, particularly for perforated recordings.

Anaesthetize the animal and restrain the head in stereotaxic apparatus ● TIMING 30―60 min

4| Anaesthetize animals with an initial dose of sodium pentobarbital (i.p.; 80 mg/kg for rats, 85 mg/kg for mice). If the anaesthetized recording is of interest, also make tracheotomy.
5| Restrain the head in a stereotaxic apparatus. Keep the body temperature at 37.3―37.8 °C with a heating blanket.
6| Shave the fur on the top of the animal’s head.
7| Cut the scalp to expose the skull and clean completely the soft tissue above the skull.
8| Proceed to Step 9 for anaesthetized recordings; follow Step 8 (i) to 8 (v) for awake recordings (in head-fixed mice) ● **TIMING 20―30 min for (i) and 40―60 min per day for (iii and iv)**

**Fig. 9:**
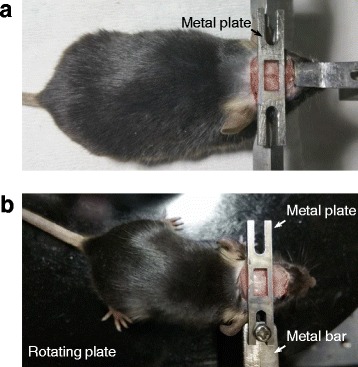
Animal preparation for awake, head-fixed recordings. (**a**) A metal plate was attached on the mouse skull under anaesthesia. (**b**) The animal was placed on a rotating plate (plastic) with the metal plate on the skull screwed to a metal bar (installed laterally on one side of the head) to get accustomed to head fixation
(ii) After attaching the metal plate, house mice individually.
(iii) One or two days later, train the animal to get accustomed to head fixation in the recording setup, by placing animals on a plastic, rotating plate and mounting the metal plate attached on the skull to a metal bar (the metal bar can be installed laterally on either side of the head) (Fig. 9b). After 40―60 min training, return the animal to home cages.
(iv) Repeat the training described in (iii) for 3 or 4 more days. (See Additional file 1: Supplementary Movie for a mouse that had been accustomed to head fixation).
(v) On the recording day, anaesthetize mice with isoflurane (1.5 %) and in the same way as described in (iii), fix the head in the recording setup.

(i) Attach a metal plate (which allows the exposure of Bregma and craniotomy) on the mouse skull with Vetbond tissue adhesive and dental acrylic (see Fig. 9a; the metal plate was customized).

Additional file 1: Supplementary Movie. (MP4 2935 kb)

Craniotomy and dissection of the dura without or with removing subdural meninges ● TIMING ~15 min for dissecting only the dura and 50―100 min for dissecting both the dura and subdural meninges

9| Under anaesthesia, make a small craniotomy (2―3 mm diameter for rats, 1―1.5 mm for mice) for recordings at the site of interest (in our experiments, 6―8 mm posterior and 3―4.5 mm lateral from Bregma for visual cortical recordings in rats; 3.5―5.5 mm posterior and 2.5―3.5 mm lateral from Bregma for recordings in the rat hippocampus; 1.5―2.5 mm posterior and 1.5―1.9 mm lateral from Bregma for recordings in the mouse hippocampus). Make another craniotomy (~0.8 mm diameter) for placement of an Ag/AgCl reference electrode.
10| Follow option (A) if dissecting only the dura is of interest and follow option (B) if dissecting both the dura and subdural meninges is of interest.

(**A**) Dissecting the dura without any mechanical damage to subdural meninges.

(i) ▲CRITICAL Carefully select a tungsten needle that has a sharp (<3 μm) and stiff tip (see Fig. 1a). Note that the needle with a blunt tip is hard to avoid touching subdural meninges during dissection of the dura, and the needle with a sharp tip but not stiff enough cannot firmly hook up the dura to remove it. We also found that a stainless steel needle with such a sharp tip is not stiff enough.
(ii) Use the tungsten needle to hook up the dura and remove a small piece (0.2―0.5 mm) of the dura, during which subdural meninges should not be touched by the needle (as shown in Fig. 1b, c). After dissection, keep the exposed brain surface moist with saline.

▲CRITICAL This is one of the most critical steps. Any touch to subdural meninges by the needle (in our experiments, even without any visible damage under the dissection microscope at ⨯63 magnification) make the whole-cell recording (particularly the perforated recording) very difficult to be achieved.

(iii) When needed, use a fine forceps for further dissection of the dura, but do not touch subdural meninges.

(**B**) Dissecting both the dura and subdural meninges.

(i) Remove most of the dura within the craniotomy with a metal needle and fine forceps. Unlike in option (A), it is not necessary to avoid toughing subdural meninges by the needle, and blunt needles (tip, 5―15 μm; tungsten or stainless steel) can be used.
(ii) At the location for pipette penetration, dissect completely subdural meninges with the use of the needle and fine forceps. When bleeding occurs, wait for the stop of bleeding to repeat dissection. After dissection, keep the exposed brain surface moist with saline.

▲CRITICAL The subdural meninges above the cortical surface for pipette penetration should be completely removed. Any remaining subdural meninx makes patch recordings (particularly perforated recordings) very difficult to be achieved.

? TROUBLESHOOTING (2)

11| For recordings from awake, head-fixed animals, release the head after surgery (in Step 9 and 10) and wait for (>1 hr) animals to recover from isoflurane anaesthesia (in Step 8 (v)). Then, re-fix the head for 4―5 hr for achieving recordings, during which water can be applied (1 to 3 times). For recordings from anaesthetized animals, keep the anaesthesia at a light level that is just below the threshold of body movement consisting of licking or scratching, for which supplementary injection of pentobarbital (16―20 mg/kg/hr in our experiments from rats; with the use of a syringe pump) should be conducted 2―4 hr after initial anaesthesia.

Fill recording pipettes and clean the brain surface ● TIMING 5―10 min and 2―5 min for perforated and conventional patch recordings, respectively

12| Follow option (A) for perforated whole-cell recordings and follow option (B) for conventional whole-cell recordings.

(**A**) Adding glass beads in internal solution for perforated recordings and pipette filling.

(i) Add a small amount (0.5―0.8 mg/ml) of glass beads (5―15 μm in diameter) in amphotericin-containing internal solution (see Reagent setup) and within 5―10 min before pipette filling, evenly mix the glass beads with solution by placing the centrifuge tube that contains internal solution on a vortex mixer for 10―20 sec.
(ii) Fill the internal solution that contains both amphotericin and glass beads in a recording pipette.
(iii) Insert the recording pipette in its holder of a pipette pressure system (which is not necessary to be the one for recording) that is mounted on an inverted microscope. Immerse the pipette tip in internal solution that is added in a 35 ⨯ 10 mm culture dish, and visualize the tip at ⨯400 magnification.
(iv) Apply a relatively low (200―300 mbar) positive pressure in the pipette interior to push glass beads to the pipette tip. If any glass bead, too small or too big, enters at a wrong position in pipette tips (the contact with the inner wall of pipette tips by the bead is not within 15―30 μm from the tip opening), apply negative pressure to suck it back and repeat the application of low positive pressure. If one or several glass beads can enter at the right position in the pipette tip (within 15―30 μm from the tip opening, as displayed in Fig. 3b) without contamination of the tip opening by precipitate of amphotericin solution, use this pipette for perforated recordings. If precipitate enters the tip opening, apply higher positive pressure (which can be gradually increased) to try to push it away from the pipette or apply negative pressure to try to suck it back (if the precipitate can be removed from the tip opening, repeat the application of low positive pressure to try to push glass beads to the pipette tip; otherwise, discard this pipette).

In about 1/2 to 2/3 pipettes, glass beads can successfully enter at the right position of their tips, and this step typically takes several minutes.

? TROUBLESHOOTING (3)

(**B**) Fill internal solution in recording pipettes for conventional whole-cell recordings.

13| In the recording setup, insert the recording pipette in its holder of the pipette pressure system.
14| Gently clean the brain surface within craniotomy with warm (32―37 °C) saline by using a small piece of cotton. If only the dura is dissected and the surface of subdural meninges has not been cleaned for > 1―2 hr, use the sharp tungsten needle for dura dissection to hook up an edge of the remaining dura to remove some secreta (which are very sticky and possibly secreted from subdural meninges) before cleaning the brain surface.

▲CRITICAL This is important for avoiding contamination of the recording pipette as it penetrates in the brain.

Achieve whole-cell recording ● TIMING variable

15| Within 10 sec after cleaning the brain surface, apply positive pressure to the pipette back (250―350 mbar and 60―80 mbar for perforated and conventional patch recordings, respectively) and lower down the pipette into brain tissues (<50 μm beneath the cortical surface) at a speed of 30―50 μm/s. Monitor pipette resistance in voltage-clamp mode (at 0 mV) by applying positive current pulses (5 mV for 10 ms at 50 Hz).
16| Advance the pipette in the brain at a speed of 15―30 μm/s (for both perforated and conventional patch recordings). When the pipette arrives at the depth of interest, use a slow speed (10―15 μm/s; for both perforated and conventional patch recordings) to advance it.
17| When reproducible increase of pipette resistance is detected (30―50 % and 20―30 % in perforated and conventional patch recordings, respectively; in perforated recording, AP-like inward currents should also occur, as shown in Figs. 4a, c and 6a), stop advancing pipettes and apply gentle negative pressure (by the mouth or a syringe) for seal formation and then hyperpolarize the pipette potential to –70 mV to further facilitate seal formation. For conventional patch recording, apply brief strong suction to rupture the cellular membrane. If the whole-cell configuration is not achieved or the whole-cell recording does not meet quality criteria, retract the recording pipette (Step 19) and repeat the procedures starting from Step 12 with a new pipette.

Under anaesthesia, the success rates to obtain perforated patch recordings and conventional patch recordings (in the neocortex or hippocampal CA1 pyramidal layer) should be no lower than 70 % and 50 %, respectively, as noted in the main text. A similar or not significantly lower success rate should be seen in recordings from awake, head-fixed animals, as found in our awake perforated patch recordings from mice.

? TROUBLESHOOTING (4)

18| In current-clamp or voltage-clamp mode, record the activity of the whole-cell patched neuron.

As noted in the main text, about 70 % perforated patch recordings can last for > 1 hr under anaesthesia, and in awake, head-fixed mice, about 50 % perforated patch recordings can last for > 1 hr. About 60 % conventional patch recordings conducted under anaesthesia can last for > 1 hr.

? TROUBLESHOOTING (5)

19| If no or no more recording is needed, retract the pipette at an initial speed of 15 μm/s for a distance of ~200 μm and then at a speed of 30―45 μm/s to the brain surface.
20| Measure the stereotaxic coordinates of the cell successfully recorded.

? TROUBLESHOOTING

**Step 3**, when the pipette pressure system is not sealed tightly enough, try to replace some elements. Based on our experience, some elements (particularly the 1-ml syringe for applying positive pressure, the three-way Stopcock, and the cone washer in the pipette holder) should be frequently replaced. It is highly recommended to compare the elements produced by different manufacturers as well as to compare and carefully select the elements even if they are produced by the same manufacturer.

**Step 10 (B)**, as a result of bleeding, dissecting subdural meninges often causes cell death in the cortex within the craniotomy (particularly in layer II/III). If severe cell death cannot be avoided (the survival of cells can be assessed by recording extracellularly spontaneous neuronal firing), try younger animals. This is because younger animals have thinner subdural meninges. In our observations in the primary visual cortex of the rat aged 10―12 weeks, cell death was not caused by dissecting subdural meninges in layer IV/V but in about 1/2 animals caused in layer II/III; in rats aged ~14 weeks, cell death usually could not be avoided in II/III and in about 1/3 animals was caused in IV/V. However, various cortical areas may exhibit different extents of cell death, due to the difference in the density of blood vessels existing in their cortical surfaces.

**Step 12 (A)**, if glass beads tend to stick together in internal solution (as observed in pipette tips under the inverted microscope after the application of air puff), they may need to be dried before adding in internal solution, for which the container (the whole bottle) of the purchased glass bead can be placed in a drying oven at 40 °C for > 10 hr. This can help the entering of a small number (one or several) of glass beads in the pipette tip (note that the entering of too many beads in pipette tips is not suitable for achieving recordings).

**Step 17**, when it is difficult to achieve high-quality recordings, check the following possibilities:

(i) Whether the dissection of the dura or subdural meninges is strictly performed according to that described in Step 10.
(ii) For cortical recordings with subdural dissected (the cortical neuron under craniotomy could die from dissecting subdural meninges due to bleeding), try to record neuronal firing with loose-patch recording or extracellular recording to assess cell survival (in our observations, nearly all whole-cell patched neurons in the sensory cortex exhibited spontaneous firing).
(iii) Whether the pipette pressure system used for recordings is sealed tightly enough, as described in Step 2 and 3.
(iv) Whether the cortical surface is well cleaned before lowering down the recording pipette in the brain, as described in Step 14.
(v) Ensure that the speeds for lowering down the pipette to the brain surface and for advancing the pipette in the brain (as recommended in Step 15 and 16) are used. Note that if any one of these speeds is too fast, the pipette would become contaminated by the brain tissue.
(vi) For rapid amphotericin perforation, amphotericin powder should not be used > 1 year after purchase. The stock solution of amphotericin (in DMSO, see Reagent setup) should not be used > 1 week after preparation, and the working solution should not be used > 0.5―1 hr after preparation (also note that the two solutions should be stored in dark conditions; –20 °C for stock solution and room temperature for working solution).

**Step 18**, when the recording is not stable, check the following possibilities:

(i) Ensure that the whole-cell recording is of a high quality (as noted in the main text).
(ii) Ensure that a small craniotomy is performed, as described in Step 9.
(iii) Craniotomy in mice needs special attention, because the mouse skull is very thin and loose. This craniotomy should be very gently and slowly performed, which can effectively reduce the pulsation of the cortex that could be induced by respiration and heartbeat or body movement (during awake states); we also suggest adding saline on the skull for gentle drilling. After making craniotomy, no pulsation of the cortex induced by respiration and heartbeat and in awake, head-fixed subjects induced by body movement (e.g., licking, grooming, and running) should be seen under the dissection microscope at ⨯63 magnification.
(iv) For awake recordings in head-fixed animals, ensure that the metal plate is tightly attached on the skull with Vetbond tissue adhesive and dental acrylic (Step 8 (i)). Under the dissection microscope (at ⨯63 magnification), no movement of the skull should be seen when the head is fixed and there is body movement (e.g., licking, grooming, and running).
(v) For anaesthetized recordings, if there is sputum in animal’s trachea, suck it out before recording (from the tube inserted via the tracheotomy described in Step 4), and select the animal (healthy and not getting a cold) with less sputum for recordings.

## Abbreviations

AP: action potential
LSD: larger and slower depolarization
RP: resting potential
